# HCV 3a Core Protein Increases Lipid Droplet Cholesteryl Ester Content via a Mechanism Dependent on Sphingolipid Biosynthesis

**DOI:** 10.1371/journal.pone.0115309

**Published:** 2014-12-18

**Authors:** Ursula Loizides-Mangold, Sophie Clément, Alba Alfonso-Garcia, Emilie Branche, Stéphanie Conzelmann, Clotilde Parisot, Eric O. Potma, Howard Riezman, Francesco Negro

**Affiliations:** 1 Division of Clinical Pathology, University Hospital, University of Geneva School of Medicine, Geneva, Switzerland; 2 Divisions of Gastroenterology and Hepatology, University Hospital, University of Geneva School of Medicine, Geneva, Switzerland; 3 Department of Biochemistry, NCCR Chemical Biology, University of Geneva, Geneva, Switzerland; 4 University of California Irvine, Beckman Laser Institute, Irvine, California, United States of America; University of North Carolina School of Medicine, United States of America

## Abstract

Hepatitis C virus (HCV) infected patients often develop steatosis and the HCV core protein alone can induce this phenomenon. To gain new insights into the pathways leading to steatosis, we performed lipidomic profiling of HCV core protein expressing-Huh-7 cells and also assessed the lipid profile of purified lipid droplets isolated from HCV 3a core expressing cells. Cholesteryl esters, ceramides and glycosylceramides, but not triglycerides, increased specifically in cells expressing the steatogenic HCV 3a core protein. Accordingly, inhibitors of cholesteryl ester biosynthesis such as statins and acyl-CoA cholesterol acyl transferase inhibitors prevented the increase of cholesteryl ester production and the formation of large lipid droplets in HCV core 3a-expressing cells. Furthermore, inhibition of *de novo* sphingolipid biosynthesis by myriocin - but not of glycosphingolipid biosynthesis by miglustat - affected both lipid droplet size and cholesteryl ester level. The lipid profile of purified lipid droplets, isolated from HCV 3a core-expressing cells, confirmed the particular increase of cholesteryl ester. Thus, both sphingolipid and cholesteryl ester biosynthesis are affected by the steatogenic core protein of HCV genotype 3a. These results may explain the peculiar lipid profile of HCV-infected patients with steatosis.

## Introduction

Hepatitis C virus (HCV) infects about 2.8% of the global population [1] and is a major cause of chronic liver disease and hepatic and extrahepatic mortality worldwide [2]. HCV interferes with lipid metabolism, at several non-exclusive levels, favouring its own replication and virion production. Occasionally, these physiopathological alterations may lead to steatosis, a feature shared by the metabolic syndrome. Fatty liver is observed in up to 80% of chronic hepatitis C patients and occurs in hepatitis C at a frequency that is more than two-fold higher compared to the general population or even to patients with other viral liver diseases, such as chronic hepatitis B [3]. This suggests that HCV may directly cause the appearance of large lipid droplets (LD) in hepatocytes. Interestingly, in patients with HCV genotype 3a, steatosis is not only more frequent and severe, but its score correlates with the HCV replication level [4]. In addition, steatosis, induced by HCV genotype 3a, disappears in the case of successful antiviral therapy. Moreover, studies in cultured cells, transfected with the HCV core protein of different genotypes, indicated that this viral protein is sufficient to induce the appearance of large LD within the cytoplasm of hepatocytes, and that the core protein of genotype 3a is by large the most efficient to induce this phenomenon [5]. Thus, although all HCV genotypes interfere with lipid metabolism, steatosis is more frequent and severe upon genotype 3 infection, suggesting that this viral genotype brings about additional perturbations in the cell biology of the host. The mechanisms underlying the variable efficiency, whereby the different viral genotypes cause the appearance of very large fat droplets in hepatocytes have been poorly characterized, and a direct comparison between different genotypes has been rarely carried out using the same experimental models [6]. For example, HCV impairs lipoprotein secretion from hepatocytes. Indeed, serum levels of apolipoprotein B (ApoB) and cholesterol are reduced in chronic hepatitis C, especially in patients with steatosis and genotype 3: successful antiviral therapy results in the correction of these anomalies [7]. However, comparable phenomena have been reported in patients with genotype 1 [8], [9]. Similarly, HCV interferes with an important factor involved in the very-low density lipoprotein (VLDL) assembly, the intrahepatic microsomal triglyceride transfer protein (MTTP). *MTTP* mRNA levels are reduced in chronic hepatitis C patients with steatosis and genotype 3 [10], although a reduced activity of this rate-limiting enzyme has been reported also in transgenic mice, constitutively expressing a genotype 1 core protein [11]. An increased *de novo* synthesis of fatty acids, through activation of the sterol regulatory element binding protein-1c (SREBP-1c), a transcription factor involved in fatty acid neosynthesis, has also been described [12], [13], although the *in vivo* data are inconclusive [14]. In addition, fatty acid oxidation is decreased via the downregulation of the peroxisome proliferator-activated receptor α (PPARα) both *in vitro* [15] and in the liver of chronic hepatitis C patients, especially – but not exclusively – if infected with genotype 3 [16], [17]. Finally, we have recently demonstrated a role for the phosphatase and tensin homolog (PTEN) in the pathogenesis of steatosis caused by HCV of genotype 3 but not 1 [18].

In this report, we aimed to further characterize the mechanism whereby HCV genotype 3 induces the formation of large LDs. To achieve this goal, we performed extensive lipidomic analysis of both total cell extracts and purified LDs isolated from Huh-7 cells, overexpressing the core protein of HCV genotype 2a (non-steatogenic) or 3a (steatogenic) [5], [19], [20]. Here, we show that the HCV 3a core protein specifically increased levels of cholesteryl ester (CE) and ceramides (Cer) and to some extent also of glycosylceramides (GlcCer). In addition, the isolated LDs contained increased amounts of ether lipids compared to whole cell lysates. Thus, steatosis induced by HCV genotype 3 is not only characterized by an increased synthesis of CE, but also by the unusual elevation of sphingolipids, whose significance warrants further investigations.

## Materials and Methods

### Primers, antibodies, plasmids and reagents

Reagents, primer sequences, antibodies and plasmids used are described in S1 Table.

### Cell culture, lentivector transduction and siRNA transfection

Human hepatoma Huh-7 cells were cultured in low glucose DMEM (Invitrogen Life Technologies) supplemented with 10% fetal bovine serum (FBS), 100 U/ml penicillin and 100 µg/ml streptomycin (all from Invitrogen Life Technologies). Lentivectors, expressing the HCV genotype 2a_KH-123 (HM053610.1) or 3a_452 (DQ437509) core protein as well as GFP respectively, have been previously described [21]. Lentivector particles were titered by both immunofluorescence and real-time PCR [22] and transduction conditions were optimized to achieve approximately 70% core-positive cells 48 hours after transduction, as assessed by immunofluorescence [21]. siRNA oligonucleotides, targeting human SPT (S1B Table), were transfected 24 hours prior transduction at 10 nM using Lipofectamine 2000 (Invitrogen).

### Lipid droplet isolation

LDs were isolated from Huh-7 cells by density sucrose gradient centrifugation as described [23]. Briefly, 10 million cells were scraped in ice-cold PBS, pelleted by centrifugation at 1000×g for 10 minutes and resuspended in ice-cold hypotonic lysis medium (HLM; 20 mM Tris-HCl, pH 7.4, 1 mM EDTA, Complete *Protease Inhibitor* Cocktail). After removal of the nuclei by low-speed centrifugation, the cell lysate was adjusted with sucrose (20% sucrose final), layered with ice-cold HLM containing 5% sucrose and then ice-cold HLM over the sucrose layers, and ultracentrifuged at 28,000×g for 30 minutes. The floating opaque LD fraction was collected from the top of the centrifuge tube and characterized by testing for immunoreactivity against a well-known LD-associated protein (adipose differentiation-related protein, ADRP). Contamination of the LD fraction with mitochondrial, peroxisomal and endoplasmic reticulum (ER) membrane fractions was assessed using anti-mitochondrial, anti-catalase and anti-calreticulin antibodies, respectively (see S1C Table). In addition, the LD fractions were also tested for cardiolipin (CL), an important lipid component of the inner mitochondrial membrane, by mass spectrometry based lipidomics.

### Immunoblotting

Proteins from whole cells or LD fractions were separated on 10% polyacrylamide gels and transferred onto nitrocellulose membranes (Milian). Membranes were blocked with wash buffer containing 5% skim milk (20 mM Tris-HCl, pH 7.4, 140 mM NaCl, 0.1% Tween 20) and incubated with primary antibodies (S1C Table), diluted in wash buffer containing 3% BSA. Membranes were further incubated with peroxidase-conjugated goat anti-mouse or goat anti-rabbit antibodies (S1D Table) diluted 1:10,000 in 0.1% BSA wash buffer. Proteins were revealed by chemiluminescence (ECL, Amersham Biosciences). Membranes were scanned by densitometry using ImageQuant *LAS 4000* (GE Healthcare Life Sciences).

### Indirect immunofluorescence and confocal microscopy

Cells were fixed in 4% paraformaldehyde for 10 minutes at RT and permeabilized with 0.2% Triton X-100 for 2 minutes. Cells were first incubated with anti-HCV core antibody (S1C Table), diluted in PBS-Tween 0.1% for 30 minutes at RT. Samples were then incubated with Alexa488-conjugated anti-mouse antibodies (S1D Table) and DAPI for nuclear staining, for 30 minutes at RT. Neutral lipids were stained with Oil Red O (ORO). Images were acquired with a confocal microscope (LSM700, Zeiss). The surface area of LD was calculated using the Metamorph software (Molecular Devices Corporation).

### RNA isolation, reverse transcription and real-time PCR

Total RNA was extracted using the RNeasy Mini Kit (Qiagen). cDNA was synthesized from 1 µg total RNA with SuperScript II RNase H(-) reverse transcriptase (Invitrogen Life Technologies) and random hexanucleotides. Relative quantification was performed by real-time PCR as described [24] using the specific primers listed in S1A Table; primers targeting eukaryotic elongation factor 1 A-1 (*EEF1A1*) and beta-glucuronidase (*GUSB*) were used as references for normalization.

### Lipidomics

#### Lipid extraction protocols

Lipid extracts were prepared using methyl-tert-butyl ether (MTBE) as previously described [25]. Briefly, 2.5×10^6^ cells were resuspended in 100 µl water and 360 µl methanol. A mix of internal standards (S1F Table) was added (400 pmol DLPC, 1000 pmol PE31:1, 1000 pmol PI31:1, 3300 pmol PS31:1, 2500 pmol C12SM, 500 pmol C17Cer and 100 pmol C8GC, Avanti Polar Lipids). Samples were vortexed and 1.2 ml MTBE was added. Phase separation was induced by adding 200 µl of MS-grade water. The upper organic phase was transferred into a 13 mm glass tube (Corning) and the lower phase was reextracted with 400 µl of artificial upper phase [25]. To prepare lipid extracts from LDs, the sucrose gradient LD fraction was harvested and corresponding volumes of methanol and MTBE were added. The amount of artificial upper phase was calculated accordingly. The total organic phase, recovered from each samples, was split into three parts and dried. One part was treated by alkaline hydrolysis [25] to enrich for sphingolipids and the other two aliquots were used for glycerophospholipid/phosphorus assay and sterol analysis, respectively. The determination of total phosphorus was performed as described [25].

#### Phospho-and sphingolipid analysis by tandem mass spectrometry

We performed MS-MS for the identification and quantification of phospho- and sphingolipid species, using multiple reaction monitoring (MRM), on a TSQ Vantage Triple Stage Quadrupole Mass Spectrometer (Thermo Fisher Scientific), equipped with a chip-based robotic nanoflow ion source (TriVersa NanoMate, Advion Biosciences) as described [25]. Each individual ion dissociation pathway was optimized with regard to collision energy. Lipid concentrations were calculated relative to the corresponding internal standards and then normalized to the total phosphate content of each total lipid extract. For LDs, normalization to phosphate was not feasible and data were calculated as Mol%.

#### Triglyceride and cholesteryl ester measurements

Triglycerides (TGs) were extracted using a modified Folch procedure: methanol/chloroform (1/2 v/v) were added to dried cell pellets. After 1 hour incubation at RT, 250 µl of H_2_O were added prior to centrifugation at 2000 rpm for 25 minutes. The lower phase was collected and dried overnight at RT. Cholesteryl esters (CEs) were extracted in chloroform/isopropanol/Triton-X100 (7/11/0.1 v/v/v). After centrifugation, supernatants were allowed to dry overnight at RT. TGs and CEs were measured using the triglycerides GPO/PAP kit (Roche) and the cholesterol/CE quantitation kit (Calbiochem), respectively and normalized by the protein content.

For the determination of cholesterol, CEs and TGs by gas chromatography–mass spectrometry (GC–MS), one aliquot of the MTBE lipid extraction was used. Briefly, dried lipid samples were resuspended in chloroform/methanol (1/1 v/v) and injected into a VARIAN CP-3800 gas chromatograph as described [26] equipped with a Factor Four capillary column and analyzed by a Varian 320-MS triple quadrupole (Agilent Technologies) with the electron energy set to −70 eV at 250°C. Cholesterol and CEs were identified by their retention times (compared to standards) and fragmentation patterns. TGs were identified by GC-MS using a method previously described [27].

### Raman spectroscopy and coherent anti-Stokes Raman scattering acquisition

Spontaneous Raman spectra from the LDs present in each cell group were acquired with a commercial Raman microscope (InVia Confocal; Renishaw). A 532 nm excitation laser was focused onto the sample with a 50×0.5NA objective. The average power at the sample plane was 22 mW. For each group, four to nine lipid droplets were investigated. The fluorescence baseline was corrected in each spectrum and the cellular background subtracted. Finally, the spectra were averaged together within each group.

Coherent anti-Stokes Raman scattering (CARS) images were obtained by combining a 1064 nm, 76 MHz mode-locked Nd:vanadate laser (PicoTrain, High-Q) and a 817 nm beam tuned from a mode-locked Ti:Sapphire laser (Mira 900, Coherent Inc.). The two beams were overlapped, both temporally and spatially, and sent into a laser scanner (FluoView 300, Olympus), attached to an inverted microscope (IX71, Olympus). The combined beams were then focused through a 20×0.75 NA objective lens (UPlanSApo, Olympus) onto the sample. The CARS signal was collected by a photomultiplier tube (R3896 PMT; Hamamatsu, Hamamatsu City, Japan) after passing a 625 nm filter with a bandwidth of 90 nm.

### Statistical analyses

All results are representative of at least 3 independent experiments. Statistical analyses were performed using an unpaired Student's *t*-test. Differences were considered significant for *p*<0.05 (*), *p*<0.01 (**) and *p*<0.001 (***).

## Results

### Effect of HCV 2a and 3a core protein on cellular triglyceride and sterol levels

**Figure 1:**
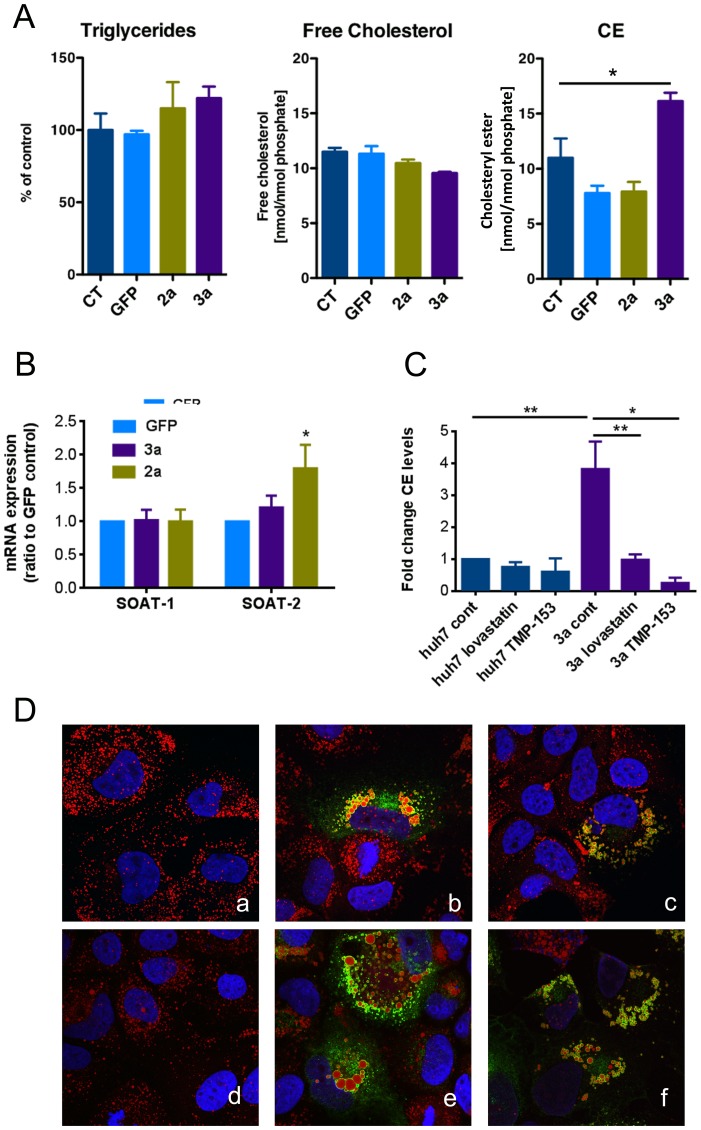
Effect of HCV 3a core on sterol and triglyceride contents and LD morphology. (**A**) Sterol and triglyceride contents were assessed by GC-MS in Huh-7 control cells (untransduced [CT]), GFP-transduced (GFP) or HCV genotype 2a or 3a core protein expressing cells. TGs, cholesterol and cholesteryl esters (CE) were identified by their retention times and fragmentation pattern. (**B**) The expression of SOAT-1 and -2 mRNA was assessed after transduction of Huh-7 cells with HCV core protein of genotype 3a, 2a or GFP at three days post-transduction by RT-qPCR. Results are represented as mean ±SEM. (**C**) Huh-7 cells were treated for 48 hours with lovastatin (10 µM) or with the SOAT inhibitor TMP-153 (25 nM) and CE levels were measured using the cholesterol/cholesteryl ester quantitation kit. Values were normalized to the amount of protein. Results are represented as mean ± SEM of at least 3 independent experiments. (**D**) Representative confocal pictures of ORO-stained Huh-7 cells either untransduced (a, d) or transduced with lentivectors expressing the core 3a untreated (b, e) and treated with 10 µM of lovastatin (c) or 25 nM TMP-153 (f) for 48 hours.

Huh-7 cells were transduced with lentivector constructs, coding for the core protein of HCV genotype 2a or 3a and compared to GFP control or untransduced cells. TG, free cholesterol and CE levels were measured by GC-MS. As shown in Fig. 1A, there was no difference in TG or free cholesterol levels between the different conditions. However, we found a strong increase in CE levels in cells expressing the genotype 3a core protein, which was not observed in cells expressing the core protein of genotype 2a or GFP (Fig. 1A, right panel). These results were further confirmed by analyzing free cholesterol, CE and TG levels using commercially available quantitation kits (S1 Fig.).

We next assessed the effect of different statins on CE content and LD morphology. Huh-7 cells, either untransduced or expressing HCV 3a core, were treated for 48 hours with 10 µM lovastatin (Fig. 1C and D), fluvastatin or pravastatin (S2 Fig.) and compared to untreated cells. While all statins have the same mechanism of action, they differ in terms of chemistry and pharmacokinetics. Lovastatin is lipophilic, whereas pravastatin and fluvastatin are more hydrophilic. CE levels were measured using the commercially available quantification kits. Cells expressing HCV 3a core displayed elevated CE levels that were significantly reduced upon treatment with the different types of statins (Fig. 1C and S2 Fig.). All three different statins reduced CE levels to a similar extent. However, in untransduced control cells, statin treatment did not have any significant effect on CE levels (Fig. 1C and S2C Fig.). The observed decrease in CE levels by statins was accompanied by a concomitant decrease of LD size, as shown by morphometric analysis of the confocal images of ORO stained LDs (Fig. 1D and S2A–B Fig.).

Next, we measured the mRNA expression levels of acyl-CoA cholesterol acyltransferase (*SOAT*)-1 and 2 that are the enzymes responsible for cholesterol esterification (Fig. 1B). HCV core 3a-expressing cells displayed a 1.8-fold increase of *SOAT*-2 expression when compared to GFP control cells. Therefore, we treated those cells with TMP-153, a SOAT inhibitor, which has been recently used in the context of HCV infection [28]. SOAT inhibition counteracted the increase of both CE level and LD size, induced by the HCV core 3a (Fig. 1C and D, S2A Fig.).

### Lipidomic profile of cells expressing HCV 2a or 3a core protein

**Figure 2:**
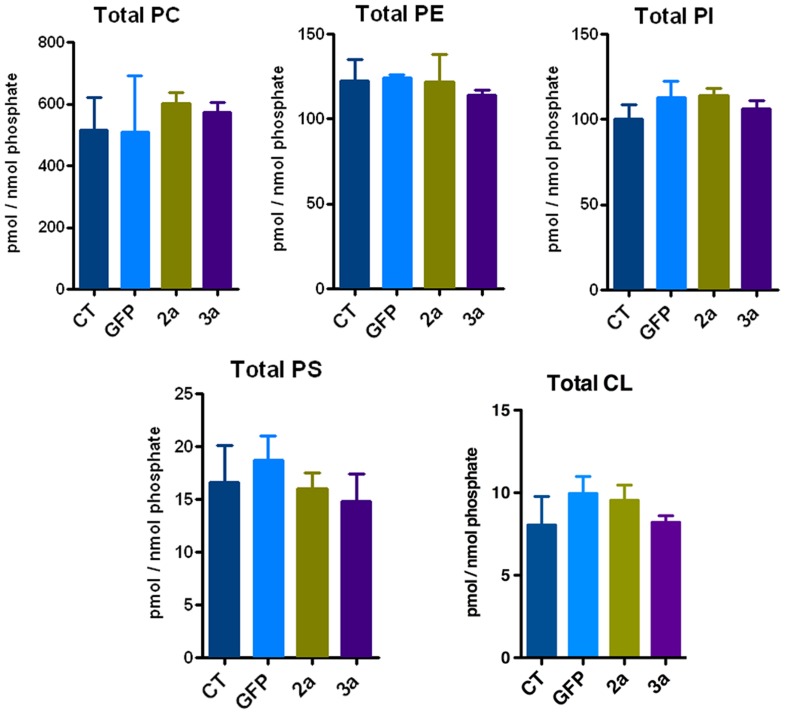
Glycerophospholipid profile of Huh-7 expressing HCV core protein. Tandem mass spectrometry was performed for the identification and quantification of glycerophospholipids. Lipid concentrations were calculated relative to the relevant internal standards and then normalized to phosphate. Total phosphatidylcholine (PC), phosphatidylethanolamine (PE), phosphatidylinositol (PI), phosphatidylserine (PS) and cardiolipin (CL) levels were determined.

Tandem MS coupled with multiple reaction monitoring (MRM) was performed to analyze the lipidomic profile of Huh-7 cells transduced with either the HCV 2a or 3a core protein, and compared to GFP control or untransduced cells. We measured the levels of>600 glycerophospholipids including phosphatidylcholines (PC), phosphatidylethanolamines (PE), phosphatidylinositols (PI), phosphatidylserines (PS) and cardiolipins (CL). Lipid metabolites were named after their total number of carbon atoms and total number of double bonds and quantified according to their corresponding internal standard. No significant differences were observed with regard to the total amount of each glycerophospholipid class (Fig. 2). Individual phospholipid profiles did not show any significant differences between 2a and 3a transduced cells (S3 Fig.). The most abundant lipid class was PC followed by lower but similar levels of PE and PI. PS and CL levels were the least abundant (Fig. 2). Each lipid class displayed a characteristic lipid profile, e.g. PC species contained lipids with shorter chain length and were overall more saturated than PE lipid species. For PC, PE, PI and PS, we also measured the related ether bond containing alkyl/acyl glycerophospholipids (abbreviated with the ending –O). Those were much less abundant than the corresponding ester linkage containing acyl/acyl glycerophospholipids (S3 Fig.).

**Figure 3:**
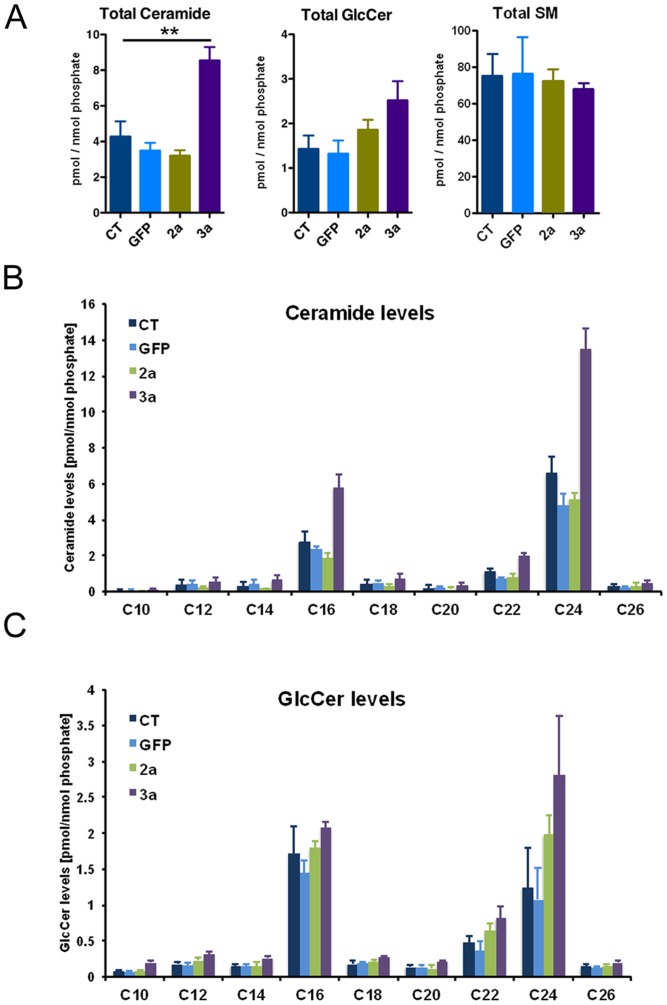
Sphingolipid profile of Huh-7 expressing HCV core protein. (**A**) Tandem mass spectrometry was performed for the identification and quantification of total ceramide (Cer), glycosylceramide (GlcCer) and sphingomyelin (SM). Lipid concentrations were calculated relative to the corresponding internal standards and then normalized to phosphate. Ceramide (**B**) and GlcCer (**C**) species were analyzed according to their fatty acid chain length.

In addition to the phospholipid profile, we also analyzed the sphingolipid profile of Huh-7 cells by assessing the levels of ceramides (Cer), glycosylceramides (GlcCer), and sphingomyelins (SM). Due to their identical mass and precursor/product ion pair *m/z* values glucosyl- and galactosylceramides cannot be distinguished by tandem mass spectrometry (MS/MS). Glucosyl- and galactosylceramide isomers are therefore referred to collectively in the text as glycosylceramides (GlcCer). Total ceramide levels were markedly increased in the HCV 3a core protein expressing cells (Fig. 3A). The increase in ceramides translated also into a modest increase in GlcCer species (Fig. 3A), whereas SM levels were not affected (Fig. 3A). Furthermore, we analyzed sphingolipid levels according to their chain length. As shown in Fig. 3B, both long chain (C16) and very long chain (C22/C24) containing ceramide species were strongly upregulated. Concerning GlcCer species, we also observed a modest overall increase in all GlcCer species (Fig. 3C).

### Ceramide levels impact lipid droplet size and cholesteryl ester levels in HCV 3a core protein transduced cells

**Figure 4:**
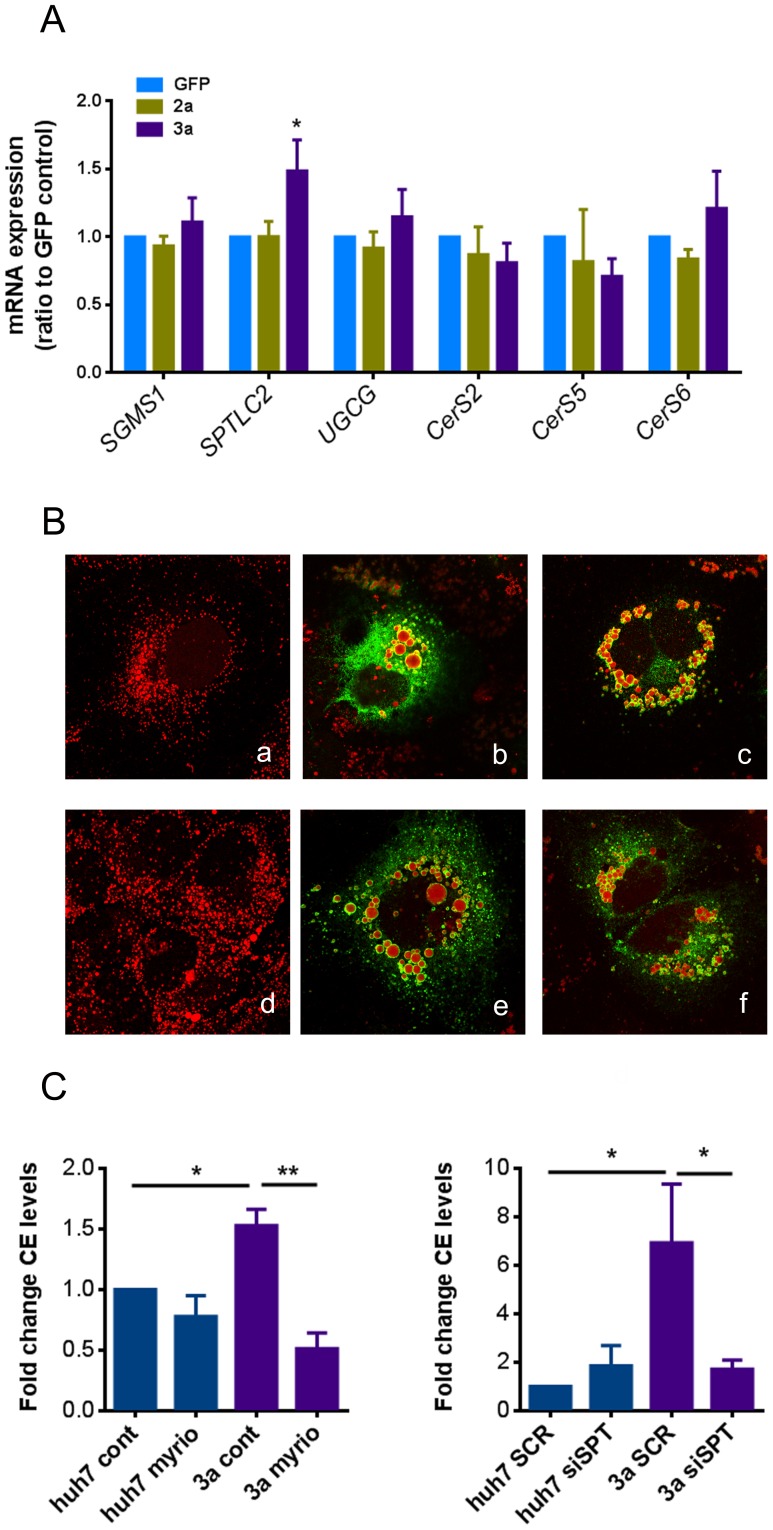
Effect of HCV core protein expression on mRNA levels of key enzymes of sphingolipid biosynthesis, cell treatment with inhibitors of sphingolipid biosynthesis and silencing of SPT. (**A**) The expression of key enzymes involved in sphingolipid biosynthesis (SGMS1: sphingomyelin synthase; SPTLC2: serine palmitoyl transferase; UGCG: glucosylceramide synthase; CerS 2, 5 and 6: ceramide synthase 2, 5 and 6) was assessed after transduction of Huh-7 cells with HCV core protein of genotype 3a, 2a or GFP at three days post-transduction by RT-qPCR. Results are represented as mean ±SEM. (**B, a–c**) Huh-7 cells were pre-treated with 200 nM myriocin for 24 hours before transduction with HCV 3a core-expressing lentivector and then treated with 200 nM myriocin for an additional 48 hours. Control cells (a), untreated core 3a transduced Huh-7 cells (b) and core 3a transduced Huh-7 cells treated with myriocin (c). (**B**, d–f) Cells were transfected with either scrambled siRNA (SCR) or siRNA targeting SPT (siSPT) 24 hours prior transduction. Control cells (d), core 3a transduced Huh-7 cells transfected with SCR (e) and core 3a transduced Huh-7 cells transfected with siSPT (f). Images show representative single optical confocal immuno-fluorescence sections of ORO (red) and anti-core 3a (green) staining. Overlay images are shown. (**C**) CE levels were measured in myriocin-treated or SPT-silenced cell conditions using the cholesterol/cholesteryl ester quantitation kit. Values were normalized to the amount of protein. Results are represented as mean ±SEM of at least 3 independent experiments.

We next wanted to assess whether levels of enzymes involved in sphingolipid biosynthesis were deregulated in HCV 3a expressing cells. The mRNA expression levels of key enzymes involved in *de novo* sphingolipid biosynthesis were assessed by RT-qPCR in either untransduced cells or in cells transduced with HCV core protein 2a, 3a, or GFP. As shown in Fig. 4A, mRNA transcription level of serine palmitoyl transferase (*SPTLC2*), the key enzyme involved in *de novo* ceramide biosynthesis, was markedly increased upon core 3a expression. High SPT activity increases sphingoid base production and consequently causes an increase in ceramide and complex glycosphingolipid synthesis. Transcription levels of sphingomyelin synthase 1 (*SGMS1*) or glucosylceramide synthase (*UGCG*) however were not affected (Fig. 4A). This result might therefore explain why HCV core3a expressing cells could accumulate ceramide without an increase in complex sphingolipids such as sphingomyelins. The expression levels of ceramide synthase (CerS)-2, -5 and -6, which specifically synthesize Cer species that are increased by HCV core 3a [CerS2 (C22, C24) and CerS5 and -6 (C16)] were likewise not modified (Fig. 4A).

Then, we treated Huh-7 cells with inhibitors of sphingolipid biosynthesis such as myriocin, a potent inhibitor of SPT, or miglustat, a specific inhibitor of UGCG. Both myriocin and miglustat had the expected effects on sphingolipids (S4 Fig.). Treatment with myriocin significantly lowered ceramide, glycosylceramide and sphingomyelin (SM) levels whereas miglustat only reduced GlcCer levels as quantified by mass spectrometry (MS/MS) (see S4 Fig.). We furthermore assessed total levels of sphinganine-1 phosphate (DHS1P) and sphingosine-1-phosphate (S1P) by MS/MS. Sphingoid base levels were not statistically changed upon myriocin treatment (S4E Fig.), which showed that treatment with myriocin reduced ceramides and complex glycosphingolipids without elevating sphingoid bases.

Interestingly, treatment with the SPT inhibitor myriocin (Fig. 4B and S2A Fig.), but not with the UGCG inhibitor miglustat (S2A Fig.) had an effect on LD size in cells expressing HCV 3a core protein, suggesting that elevated ceramide – but not GlcCer – levels impact LD size in HCV 3a core protein transduced cells. Since overexpression of the HCV 3a core protein did not affect SM levels, we attributed the effect of myriocin mainly to the reduction of ceramides.

Furthermore, we quantified the effect of myriocin on total CE levels. As shown in Fig. 4C, myriocin treatment prevented the increase of CE levels induced by HCV 3a core. In order to rule out a potential non-specific effect of myriocin, we used siRNA to silence the gene expression of SPT in Huh-7 cells transduced with HCV 3a core protein. We observed effects similar to those seen with myriocin (Fig. 4B and C and S2A Fig.), further emphasizing the importance of ceramides on LDs and indicating an interplay between sphingolipid biosynthesis and cholesteryl ester levels.

### Lipidomic profile of lipid droplets from HCV core protein overexpressing cells

The lipidomic profile of LDs, isolated by density gradient centrifugation from cells overexpressing HCV 2a or 3a core protein, was assessed and compared to the profile of LDs isolated from cells expressing GFP, untransduced or incubated with oleic acid. The LD yield was assessed by testing for immunoreactivity against ADRP (S5 Fig.). LD purity was further assessed using an anti-mitochondrial marker, anti-catalase and anti-calreticulin antibodies, demonstrating that the LD preparation was not contaminated with either mitochondrial, peroxisomal or ER fractions (S5 Fig.). LDs are composed of a neutral lipid core that consists mainly out of TGs and CEs and is surrounded by a lipid monolayer. Free cholesterol, which is a component of lipid bilayers and regulates membrane fluidity, is only found in small amounts in the lipid monolayer that surrounds the lipid droplet. The opposite is true for cholesteryl esters, which are not present in the lipid monolayer and are the main component of the lipid droplet core.

**Figure 5:**
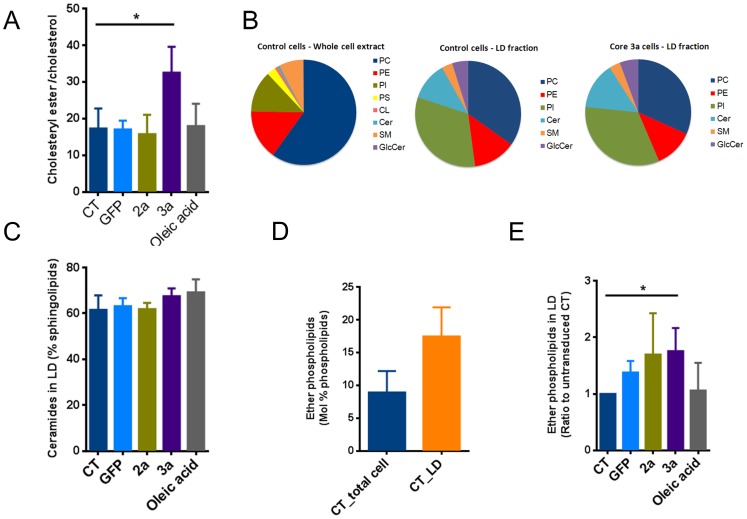
Lipid droplet lipid profile. LDs were isolated by density gradient centrifugation from Huh-7 cells transduced with HCV core protein of genotype 3a, 2a or GFP. The phospholipid profile of the LD monolayer was assessed by tandem mass spectrometry. (**A**) Ratio between cholesteryl ester and cholesterol is elevated in 3a transduced cells. (**B**) Lipidomic profile of LD isolated from either control or core 3a-expressing cells compared to the total cell lipid profile in control conditions. (**C**) Effect of HCV core expression or oleic acid stimulation on ceramide content in LDs. Data are presented as % of sphingolipids. (**D**) Total ether phopspholipids in whole cell extracts compared to LD fraction in untransduced cells. (**E**) Effect of HCV core expression or oleic acid stimulation on ether phospholipid content in LDs. Data are presented as ratio to untransduced control. Results are represented as mean ±SEM.

First, we observed an increase in the CE/cholesterol ratio in LD isolated from HCV 3a core-expressing cells compared to controls, 2a expressing cells or oleic acid stimulated cells (Fig. 5A). We then established the phospho- and sphingolipid profile of the LD faction by tandem MS. Fig. 5B shows the lipid composition of whole cell extracts compared to LDs from untransduced cells and 3a-expressing cells. We could detect PC, PE, and PI phospholipid metabolites as well as ceramide, GlcCer and SM sphingolipid metabolites in LD extracts. However, we could not detect PS and CL, further confirming the purity of our LD preparations. Importantly, the composition of the LD monolayer was markedly different from the lipid composition of whole cells extracts. Due to the low amount of phosphate in the LD preparations, normalization using the phosphate assay was not feasible and results are shown as Mol%. As shown in Fig. 5B, relative levels of PC were reduced, whereas PE and PI levels were increased. Interestingly, we also observed a substantial amount of sphingolipids in the lipid monolayer of LDs. However also in this case the profile was different from whole cell extracts as ceramide was the dominant sphingolipid in LDs compared to sphingomyelin in whole cell extracts (Fig. 5B). Interestingly, the phospho- and sphingolipid composition of the LD fraction of HCV 3a core-expressing cells was not different compared to those of control cells, also with regard to ceramide levels (Fig. 5B and C), suggesting that the increased level of ceramides, observed in whole cell extracts was not due to an enrichment of ceramides within the LD core or LD monolayer but rather caused by an overall increase in ceramides in different membrane compartments.

In addition, we found a substantial amount of ether phospholipids in the LD fractions as has been described by Bartz *et al.*, 2007 [29]. Compared to whole cell extracts, the ether lipid content was 2-fold higher in LDs (Fig. 5D). In addition, transduction with the HCV core protein but not oleic acid stimulation further increased the ether lipid content of LDs (Fig. 5E).

### LD analysis by Raman spectroscopy and CARS

**Figure 6:**
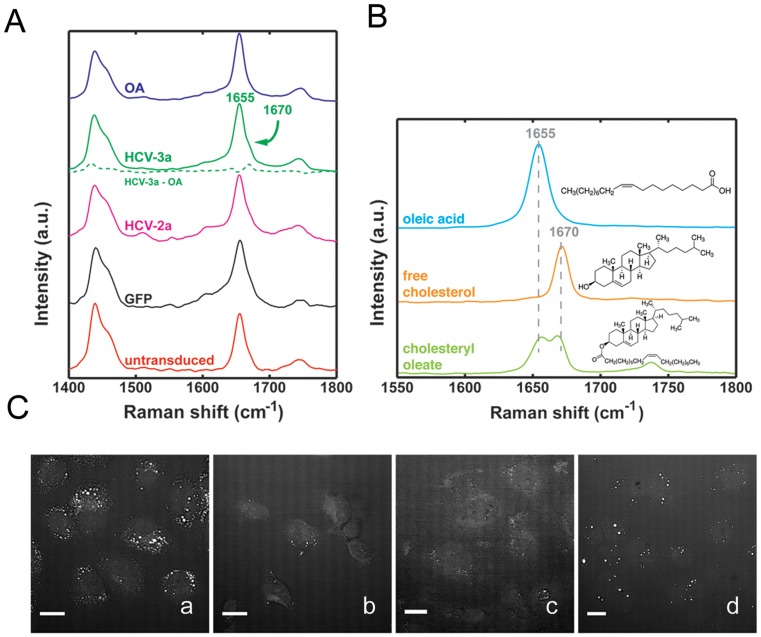
*LD analysis by Raman spectroscopy and CARS.* (**A**) Average Raman spectra (fingerprint bands) of LDs found in oleic acid loaded (OA), HCV 3a core (HCV-3a) transduced, HCV 2a core (HCV-2a), GFP or untransduced Huh-7 cells. Notice the region of interest from 1600 cm-1 to 1800 cm-1 where HCV-3a shows an extra shoulder at 1670 cm-1, besides the oleic acid peak at 1655 cm-1. This contribution is made more evident when the oleic acid contribution is subtracted from the HCV-3a spectrum, (green dotted line). (**B**) Raman spectra and molecular structure of oleic acid, free cholesterol and cholesteryl oleate. (**C**) Coherent anti-Stokes Raman scattering (CARS) images of Huh-7 cells, treated either with 200 µM oleic acid (a), untransduced (b) or transduced with lentivectors expressing core 2a (c) or core 3a (d).

We then complemented our LD lipid profiling by using Raman spectroscopy and coherent anti-Stokes Raman scattering (CARS), which allows for the visualization of lipids in cells without the need for any kind of staining. Fig. 6A shows the average Raman spectra of LDs found in Huh-7 cells that were either oleic acid-loaded, untransduced, transduced with GFP, HCV 3a or 2a core. CARS images of Huh-7 cells showed that oleic acid-loaded cells exhibited the highest number of intracellular LDs, whereas HCV 3a-transduced cells contained mostly large size lipid droplets (Fig. 6C). The Raman spectra of LDs found in cells transduced with HCV 3a core mimicked that of oleic acid-loaded cells with the exception of a small, but identifiable shoulder at 1670 cm-1 (Fig. 6A), situated besides the more prominent peak at 1655 cm^−1^. The 1670 cm^−1^ band is evident in the oleic acid/HCV 3a difference spectrum (Fig. 6A, green dotted line). This contribution is less prominent in the other groups. The 1670 cm^−1^ band, which is prominently present in the Raman spectrum of pure cholesterol (Fig. 6B), can be attributed to the C = C stretching vibration in the 6-membered cycloalkane ring of the steroid. Pure oleic acid has a strong contribution at 1655 cm-1, corresponding to the C = C stretching vibration in the unsaturated lipid chain in TGs (Fig. 6B). This peak is seen in all the groups, indicating that TGs are the major component of lipid droplets in each of the groups. The Raman spectrum of cholesteryl esters, such as cholesteryl oleate exhibits a combination of the 1655 cm-1 and 1670 cm-1 bands, producing a double-peak band structure (Fig. 6B). Thus, the presence of the 1670 cm-1 shoulder is an indication of the presence of cholesteryl ester, which appears more prominent in HCV 3a core LDs.

## Discussion

HCV infection is characterized by an unusually tight relationship with the host cell lipid metabolism. Steatosis is one among the several features that define this relationship. Although it is established that HCV induces some lipid species to benefit its own life cycle, and that LDs act as a platform for HCV particle assembly, the clinical and virological impact of steatosis – i.e. the appearance of very large LDs in the hepatocyte cytoplasm – is unclear. Since the viral core protein is sufficient to cause steatosis in experimental models, we studied in detail the lipidome of Huh-7 hepatoma cells overexpressing the HCV core protein of different HCV genotypes, i.e. the steatogenic 3a and the non-steatogenic 2a, to identify lipid changes that may affect HCV life cycle and possibly pathogenesis. We observed that the core protein of HCV genotype 3a – but not 2a – increased CE, GlcCer and ceramide levels, while it failed to increase TGs significantly, suggesting that it specifically affects CE homeostasis, thereby leading to steatosis. This result is in contrast to the common oleic acid treatment, which strongly increased TGs.

The lipid species accumulating during HCV infection and/or experimental HCV protein expression have been only partially characterized, with sometimes conflicting results. *In vitro*, the transient expression of the HCV genotype 3a and 1b core proteins led to TG accumulation [5]. In other studies, however, the core of genotype 1b, expressed in HepG2 cells [15] as well as the genotype 2a chimeric virus, (J6/JFH-1) [30] expressed in Huh-7.5 cells failed to induce TG increase. On the other hand, intrahepatic TG increase has been observed in several mouse models [15], [31]–[33]. Data are quite scarce concerning the liver lipidome of HCV-infected patients. In a very small number of chronic hepatitis C patients with steatosis, only the relative abundance of the C18:1 containing TGs fraction was reported to be slightly elevated compared to uninfected patients, while the absolute levels of liver TGs were not described [31]. At this point, it is challenging to fully explain this discrepancy in TG levels but the differences may rely on the experimental conditions used. For instance, *in vitro* experiments of HCV core expression, performed in the presence of delipidated serum, totally abrogated TG accumulation [5], suggesting that the composition of the culture medium may significantly affect the results.

In any case, if confirmed in chronic hepatitis C patients, our data showing an exclusive effect of HCV genotype 3a on CE may suggest a paradigm shift in that viral steatosis may be distinct from metabolic steatosis. Indeed, a thorough lipidomic analysis has been reported for patients with non-alcoholic fatty liver disease, showing a ∼10-fold increase of intrahepatic TG and a 2.5-fold increase of diacylglycerides compared to healthy controls, but no change in liver CE levels [34].

The relevance of the above findings to the other metabolic pathways altered by HCV and possibly leading to steatosis is unclear. We have previously shown that downregulation of PTEN by the HCV core protein leads to the appearance of large lipid droplets in human hepatoma cells, and that this effect is more important in the case of HCV 3a [18]. In this model, the downregulation of PTEN led to a reduction of insulin receptor substrate 1 (IRS1) expression, and both events seemed involved in the altered lipid metabolism leading to steatosis, since the overexpression of either PTEN or IRS1 prevented the development of large lipid droplets [18].

Statins upregulate PTEN [35], and increase both the mass and level of tyrosine phosphorylation of liver IRS-1 [36]: thus, the effect of statins reported in our work may well be mediated by actions brought about on PTEN, IRS-1 or both. PTEN lipid phosphatase function – but not absolute levels – is deeply affected by ceramides, that cause PTEN translocation to lipid rafts in neurotumoral cell lines, leading to dephosphorylation of Akt and sensitizing cells to apoptosis [37]. However, this is unlikely to be involved in the pathogenesis of steatosis, because we have shown that it is the protein, rather than the lipid phosphatase activity of PTEN that increases CE production in hepatocytes, leading to the appearance of large lipid droplets [38]. Finally the effect of myriocin on PTEN and IRS-1 regulation and function has not been investigated so far.

The fact that HCV may interact with the cholesterol synthesis pathway has been suggested previously. HMGCoA reductase, a key enzyme of the cholesterol biosynthesis pathway, was shown to be elevated in HCV patients [39] and cholesterol levels are increased in Huh-7 cells infected with HCV [40], [41]. In addition, our observation that statins, well known inhibitors of HMGCoA reductase, impede the increase of LD size caused by HCV 3a core protein, points out the crucial role that CE could play in HCV-induced steatosis as well as in HCV life cycle. Regarding the latter, while the antiviral effect of statins in chronic hepatitis C patients is inconsistent and depends on the type of statin used [42]–[45], their inhibitory effects on HCV life cycle *in vitro* has been established in several independent studies [38], [46]. A small-size randomized trial has failed to report a significant effect of simvastatin on the severity of non-alcoholic fatty liver [47], in keeping with the above mentioned lipidome study [34].

We also observed an increase in ceramide levels and to some extent as well for complex glycosphingolipids in HCV 3a core-expressing cells. When we assessed the expression of key enzymes of sphingolipid *de novo* biosynthesis, SPT was upregulated in these cells, suggesting that the HCV core protein could induce this enzyme at the transcriptional level. Whether this effect is solely dependent upon *de novo* ceramide biosynthesis or whether the ceramide salvage pathway is involved as well remains to be determined but our results reinforce the assumption that sphingolipid biosynthesis and the relatively high amount of ceramide, found in intracellular membranes, may actively contribute to the steatogenic process. Accordingly, treatment of HCV 3a core expressing-cells with myriocin (a specific inhibitor of *de novo* sphingolipid biosynthesis) prevented the LD effect. A similar effect of myriocin on steatosis has been previously shown in other models, e.g genetically obese (ob/ob) and high-fat diet-induced obese mice [48]. This observation is particularly interesting in the context of HCV infection, since inhibition of the sphingolipid metabolism has been proposed as a new strategy for treatment of HCV [49]. In a model of humanized mice (transplanted with human hepatocytes permissive to HCV), Hirata *et al.* observed an increase of sphingomyelin synthase mRNA levels but also an increase of hepatocyte ceramide and sphingomyelin contents [50]. It has been suggested that the effect of sphingolipid inhibition on HCV replication is mostly mediated by sphingomyelin and through its role in the formation of ceramide-rich membrane platforms. However, our results suggest that this effect may foremost involve ceramides via *de novo* ceramide biosynthesis. Finally, myriocin reduces HCV replication in a mouse model [51] and, even more interesting in the context of our data, a combination treatment of HCV (JFH1, genotype 2a) infected cells with myriocin and simvastatin was shown to attenuate viral replication synergistically [52]. Taken together, our results show that myriocin has not only an effect on LD morphology, but also decreases CE levels and suggest that CE and sphingolipid pathways may be interconnected. However, the exact role of ceramides in HCV core-induced steatosis remains to be determined. Although it is unlikely that ceramides serve as direct substrates for CE synthesis, they may act to stabilize/activate enzymes involved in the cholesterol pathway. Indeed, besides their role in membrane structure by altering membrane fluidity and permeability, ceramides have been shown to have signaling properties and regulate a number of enzymes including protein kinases and phosphatases involved in important biological effects (i.e, cell differentiation, cell proliferation, cell signaling) (for review see [53]). Among them, one interesting candidate could be protein phosphatase 2A [54], which has been shown to modulate the activity of HMGCoA reductase [55].

The lipidomic profiling of LD performed by both GC-MS and Raman spectrometry demonstrated that some of the above changes in CE content observed in whole cells upon HCV core 3a protein expression could also be detected in LDs. LDs are neutral lipid storage organelles. They are composed of a core of esterified lipids (mainly TGs and CEs), surrounded by a lipid monolayer membrane, in which specific proteins of the perilipin-ADRP-TIP47 (PAT) family are attached. Our data suggest that the lipid monolayer of LDs has a specific lipid composition, which was characterized by undetectable levels of PS and CL [56], and an unexpected enrichment in sphingolipids (about 20% of total, compared to the 10% found in whole cell membranes). In contrast to whole cells, ceramide and GlcCer were abundant in LDs, whereas sphingomyelin, which is the dominant sphingolipid in whole cells, was greatly reduced. However, ceramide or GlcCer levels were not increased within LDs upon HCV 3a expression, indicating that ceramides must be predominately accumulating in other cellular compartments. Due to their physical property it is unlikely that ceramides are located within the hydrophobic core of the lipid droplet. However, it is imaginable that they are converted into more hydrophobic species such as 1-O-acylceramides, which then might accumulate within the LD core. We therefore also assessed 1-O-acylceramides [57] by mass spectrometry but were not able to detect those in our LD preparation, suggesting again that ceramides are not accumulating within the LD core fraction.

The lipid monolayer of LDs (especially from cells expressing the HCV core protein) contained also high amounts of ether lipids compared to whole cell lysates. Our results are in accordance with the presence of ether lipids in LDs isolated from CHO K2 cells [29]. The enrichment of ether lipids was specific for cells expressing HCV core protein and was not observed upon oleic acid stimulation. Ether lipid biosynthesis takes place in peroxisomes and involves the peroxisomal enzymes dihydroxyacetonephosphate (DHAP) acyltransferase and alkyl-DHAP synthase. DHAP is usually reduced to L-glycerol-3-phosphate, which is the glycerol backbone required to synthesize new TGs. However, DHAP is also the substrate of the two above peroxisomal enzymes to synthesize 1-alkyl-DHAP, which in turn is used as alkyl donor during glycosylphosphatidylinositol (GPI) anchor biosynthesis, which allows anchoring of various cell surface proteins in mammalian cells. In this context, it is noteworthy that GPI-anchored proteins may have a role in the life cycle of some viruses such as Dengue virus [58]. Even though the relevance of such protein modification in HCV life cycle is unknown, it is interesting to note that circulating lipoviroparticles (LVP) contain ether lipids, as shown by the recent lipidomic analysis of HCV particles [59]. Furthermore, recent data point to a direct transfer of fatty acids across organellar boundaries that are in close proximity and suggest coupling of LD metabolism with peroxisomal fatty acid oxidation. Studies in both yeast *Saccharomyces cerevisiae* and mammalian cells provide strong evidence that peroxisomes interact closely with LDs [60], [61].

In conclusion, we have found that the expression of the steatogenic HCV 3a core protein induces the accumulation of CE (but not TG) in large sized LDs, and that HCV 3a core expression also affects ceramide and GlcCer lipid levels. Although ceramides did not accumulate in LDs directly, they promote CE increase, possibly in form of ceramide signaling, providing new mechanistic insights into HCV-induced steatosis.

## Supporting Information

S1 Fig **Measurement of sterol and triglyceride contents by commercially available kits.** Sterol (**A**) and TG (**B**) contents in GFP-transduced cells or Huh-7 cells expressing the HCV genotype 3a core protein were assessed using the cholesterol/cholesteryl ester and the GPO/PAP kit quantitation kit, respectively. Cells treated with 250 µM oleic acid were used as positive control. Values were normalized with the amount of protein. Results are represented as mean ±SEM of at least 3 independent experiments.
(TIF)

S2 Fig **(A) Quantification of individual LD size.** LD size was estimated using Metamorph software. Either control or HCV core 3a-expressing cells were treated with statins, inhibitors of sphingolipid (SL) biosynthesis, SOAT inhibitor (TMP-153) or were silenced for SPT. LDs with size over 100 nm^2^ were plotted. (**B**) Representative confocal pictures of ORO-stained Huh-7 cells either untransduced (a) or transduced with lentivectors expressing the core 3a untreated (b) and treated with 10 µM of fluvastatin (c) or pravastatin (d). (**C**) Huh-7 cells were treated for 48 hours with pravastatin or lovastatin (10 µM) and CE levels were measured using the cholesterol/cholesteryl ester quantitation kit. Values were normalized to the amount of protein. Results are represented as mean ± SEM of at least 3 independent experiments.
(TIF)

S3 Fig **Glycerophospholipid profile of Huh-7 expressing HCV core protein.** Tandem mass spectrometry was performed for the identification and quantification of glycerophospholipids and cardiolipins. Lipid metabolites were named according to their total number of carbon atoms and total number of double bonds and quantified according to their corresponding internal standard. Ether linked phospholipids are characterized by the suffix –O. Analysis of individual phosphatidylcholine (**A**), phosphatidylethanolamine (**B**), phosphatidylinositol (**C**), phosphatidylserine (**D**) and cardiolipin (**E**) species are shown.
(TIF)

S4 Fig **Effect of myriocin and miglustat on sphingolipid biosynthesis.** (**A**) Simplified scheme of the spingolipid biosynthesis pathway indicating the enzymes specifically targeted by the inhibitors used in the study. Effect of both myriocin and miglustat on ceramide (**B**), GlcCer (**C**), SM (**D**) and sphingosine-1-P (**E**) levels.
(TIF)

S5 Fig **Characterization of the LD fraction.** Immunoreactivity of the total cell lysate (lane 1) and LD fraction (lane 2) for anti-calreticulin, anti-catalase, anti-mitochondrial marker and anti-adipose differentiation related protein (ADRP) antibodies.
(TIF)

S1 Table Primers, antibodies, plasmids and reagents.
(DOCX)
