# Supplementary figures and images for: HCV 3a Core Protein Increases Lipid Droplet Cholesteryl Ester Content via a Mechanism Dependent on Sphingolipid Biosynthesis
Source: PLoS One. 2014 Dec 18;9(12):e115309. doi: 10.1371/journal.pone.0115309 (PMC4270764; doi:10.1371/journal.pone.0115309)

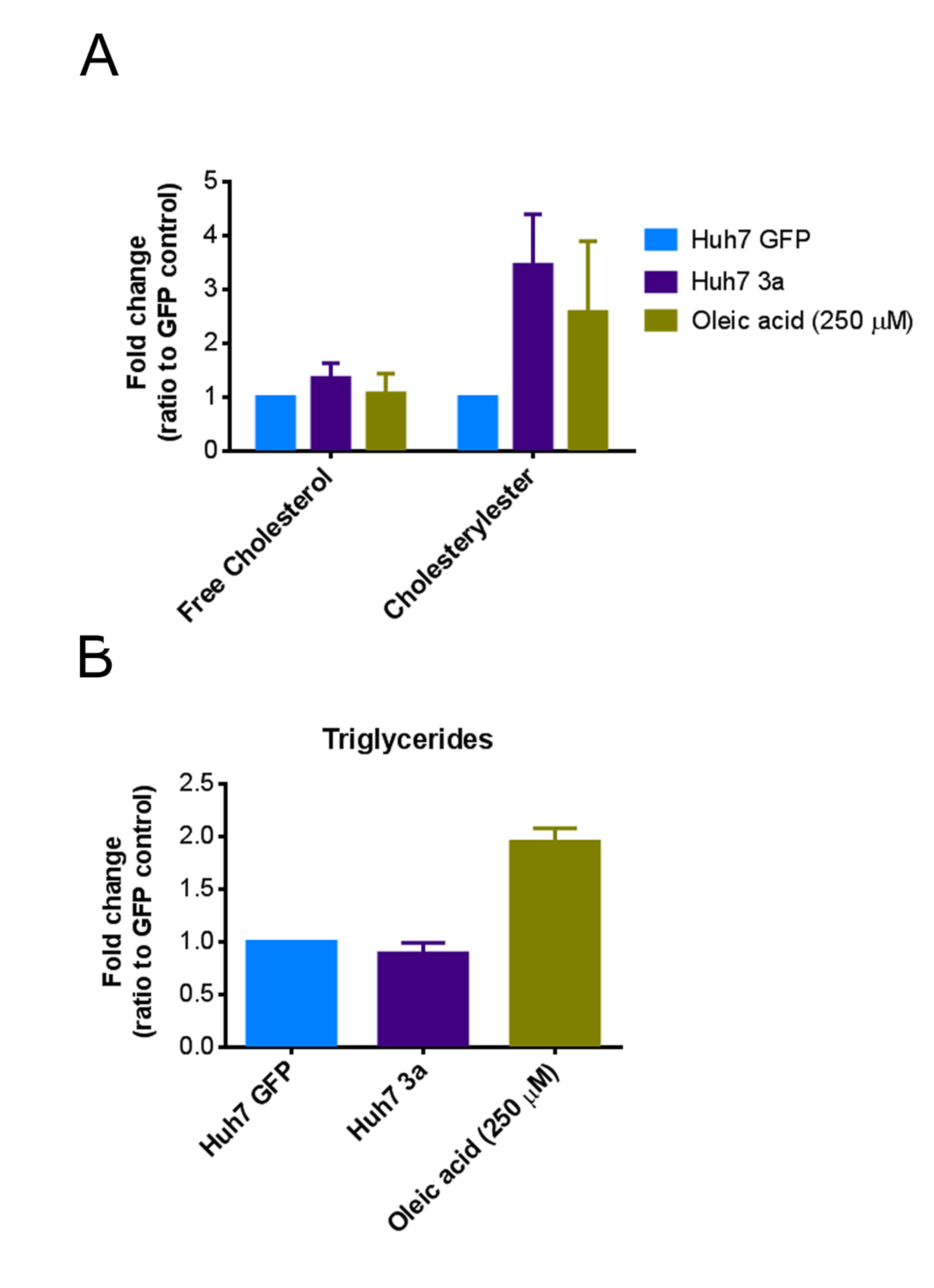

Supplement: S1 Fig — Measurement of sterol and triglyceride contents by commercially available kits. Sterol (A) and TG (B) contents in GFP-transduced cells or Huh-7 cells expressing the HCV genotype 3a core protein were assessed using the cholesterol/cholesteryl ester and the GPO/PAP kit quantitation kit, respectively. Cells treated with 250 µM oleic acid were used as positive control. Values were normalized with the amount of protein. Results are represented as mean ±SEM of at least 3 independent experiments. (TIF) [file pone.0115309.s001.tif]

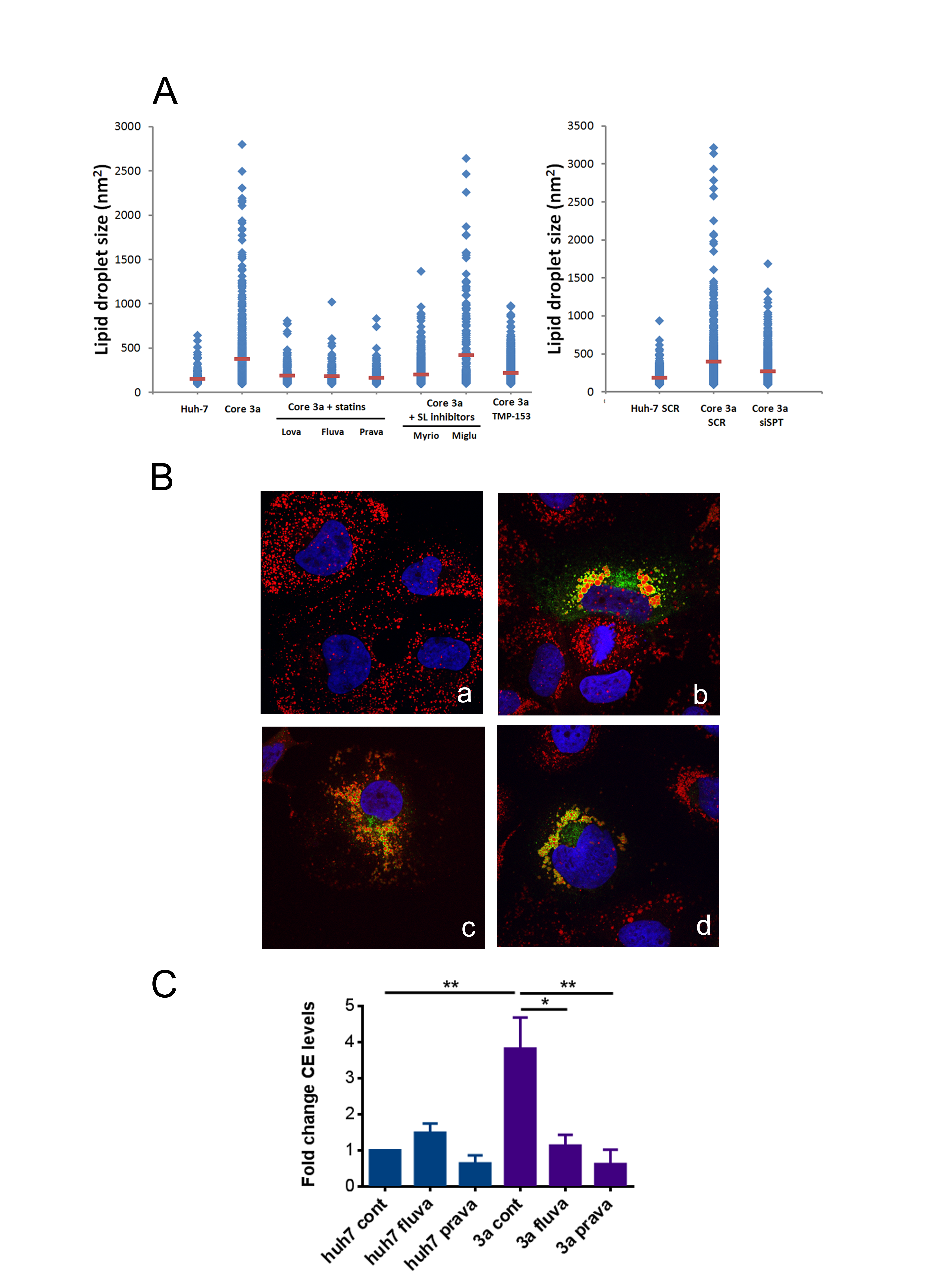

Supplement: S2 Fig — (A) Quantification of individual LD size. LD size was estimated using Metamorph software. Either control or HCV core 3a-expressing cells were treated with statins, inhibitors of sphingolipid (SL) biosynthesis, SOAT inhibitor (TMP-153) or were silenced for SPT. LDs with size over 100 nm2 were plotted. (B) Representative confocal pictures of ORO-stained Huh-7 cells either untransduced (a) or transduced with lentivectors expressing the core 3a untreated (b) and treated with 10 µM of fluvastatin (c) or pravastatin (d). (C) Huh-7 cells were treated for 48 hours with pravastatin or lovastatin (10 µM) and CE levels were measured using the cholesterol/cholesteryl ester quantitation kit. Values were normalized to the amount of protein. Results are represented as mean ± SEM of at least 3 independent experiments. (TIF) [file pone.0115309.s002.tif]

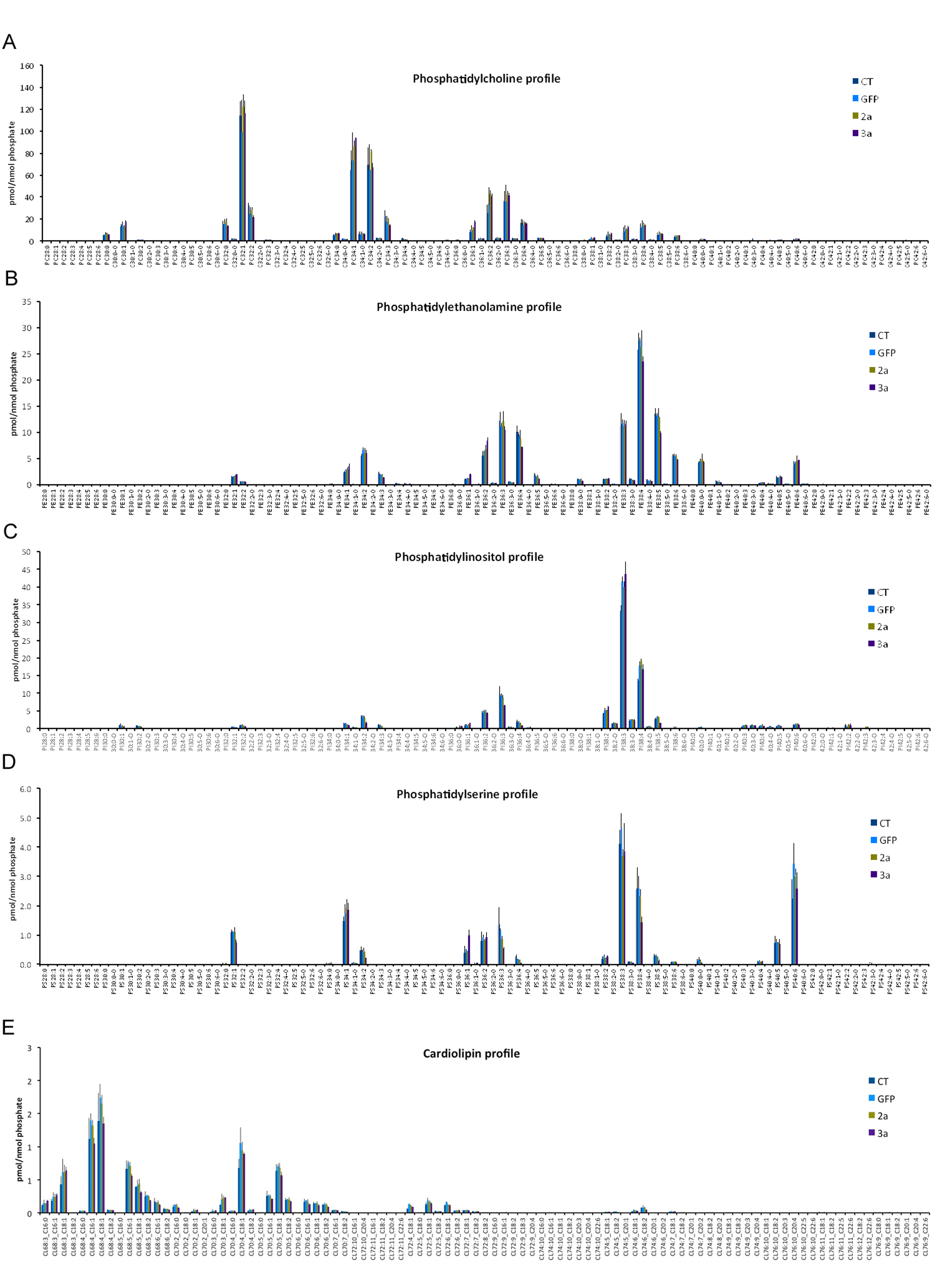

Supplement: S3 Fig — Glycerophospholipid profile of Huh-7 expressing HCV core protein. Tandem mass spectrometry was performed for the identification and quantification of glycerophospholipids and cardiolipins. Lipid metabolites were named according to their total number of carbon atoms and total number of double bonds and quantified according to their corresponding internal standard. Ether linked phospholipids are characterized by the suffix –O. Analysis of individual phosphatidylcholine (A), phosphatidylethanolamine (B), phosphatidylinositol (C), phosphatidylserine (D) and cardiolipin (E) species are shown. (TIF) [file pone.0115309.s003.tif]

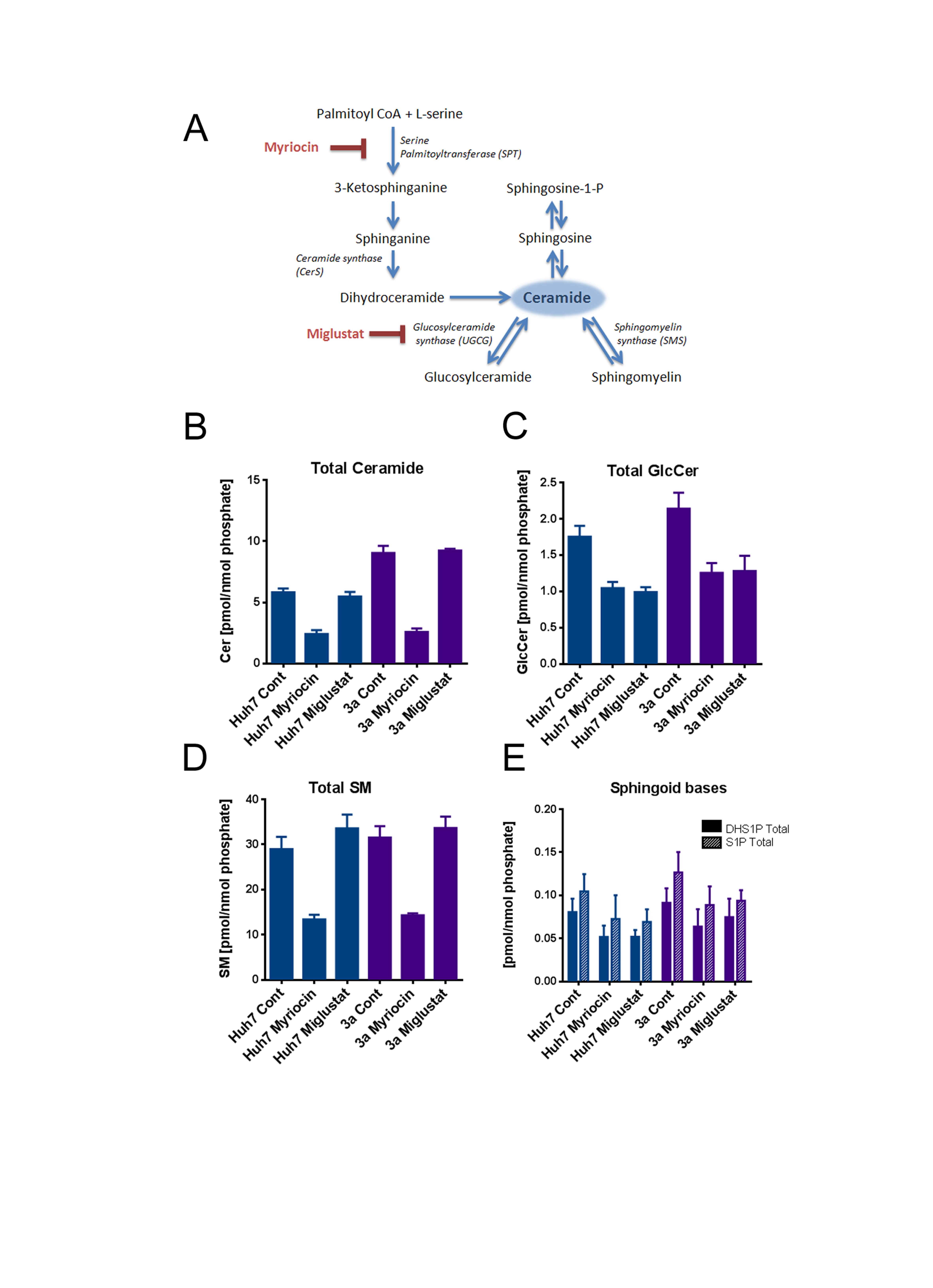

Supplement: S4 Fig — Effect of myriocin and miglustat on sphingolipid biosynthesis. (A) Simplified scheme of the spingolipid biosynthesis pathway indicating the enzymes specifically targeted by the inhibitors used in the study. Effect of both myriocin and miglustat on ceramide (B), GlcCer (C), SM (D) and sphingosine-1-P (E) levels. (TIF) [file pone.0115309.s004.tif]

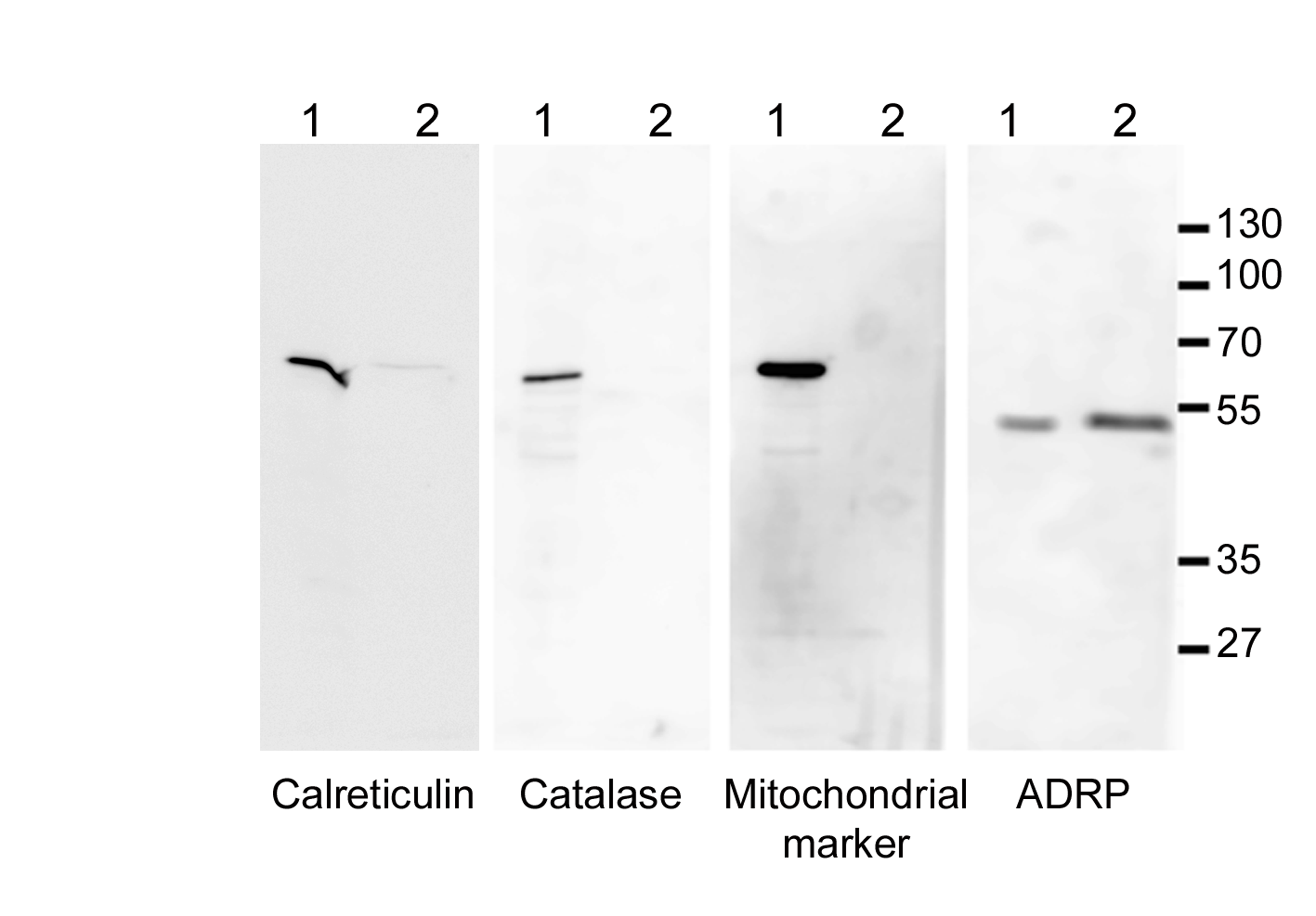

Supplement: S5 Fig — Characterization of the LD fraction. Immunoreactivity of the total cell lysate (lane 1) and LD fraction (lane 2) for anti-calreticulin, anti-catalase, anti-mitochondrial marker and anti-adipose differentiation related protein (ADRP) antibodies. (TIF) [file pone.0115309.s005.tif]
