# Supplementary material for: HCV 3a Core Protein Increases Lipid Droplet Cholesteryl Ester Content via a Mechanism Dependent on Sphingolipid Biosynthesis
Source: PLoS One. 2014 Dec 18;9(12):e115309. doi: 10.1371/journal.pone.0115309 (PMC4270764; doi:10.1371/journal.pone.0115309)
Supplement: S1 Table — Primers, antibodies, plasmids and reagents. (DOCX) [file pone.0115309.s006.docx]

**Supplementary table 1: *Primers, antibodies, plasmids and reagent***

**(A) PCR primers :**

| **Gene Name** | **Alternative name** | **Forward** | **Reverse** | **Reference** |
| --- | --- | --- | --- | --- |
| human *SGMS1* | sphingomyelin synthase | GTACCTGGTATGCATTTCAACTGTTC | TTCCCAGTCTCCGAAAAGCTT |  |
| Human  *SPTLC2* | serine palmitoyl transferase | CTGGAGGATATATTGGAGGCAAGA | GAATGTGTTCGCAGGTAGTCTATCAG |  |
| Human *UGCG* | glucosylceramide synthase | CAGGGTGGCACACTGTGTTT | GAACCAGGCGACTGCATAATC |  |
| Human CerS2 | ceramide synthase 2 | CCGATTACCTGCTGGAGTCAG | GGCGAAGACGATGAAGATGTT | (Mullen et al., 2011) |
| Human CerS5 | ceramide synthase 5 | GTTTCGCCATCGGAGGAATC | GCCAGCACTGTCGGATGTC | (Hoeferlin et al., 2013) |
| Human CerS6 | ceramide synthase 6 | ATCAGGAGAAGCCAAGCACG | AGTAGTGAAGGTCAGTTGTG | (Erez-Roman et al., 2010) |
| Human SOAT-1 | acyl-CoA cholesterol acyl transferase 1 | GAAACCGGCTGTCAAAGTCC | AATGGCTTCAATTCCTCTGC | (Read et al., 2014) |
| Human SOAT-2 | acyl-CoA cholesterol acyl transferase 2 | GAGACTTACCCTAGGACGCCC T | AGTTCTTGGCCACATAATTCCAC | (Read et al., 2014) |
| human eEF1A1 | Eukaryotic translation elongation factor 1α 1 | agcaaaaatgacccaccaatg | ggcctggatggttcaggata |  |
| human Gus B | β-glucuronidase | ccaccagggaccatccaat | agtcaaaatatgtgttctggacaaagtaa |  |
| PLV2: for lentiviral particle titration | | ACCTGAAAGCGAAAGGGAAAC | CACCCATCTCTCTCCTTCTAGCC | (Sastry et al., 2002) |

**(B) siRNA**

| **Gene Name** | **Targeted gene** | **Sequence** | **Provider** | **Catalogue Number** |
| --- | --- | --- | --- | --- |
| siSPT | serine palmitoyl transferase | TCCAGTGATTTGTCTCCGTAA | Qiagen | SI04152869 |

**(C) Primary antibodies :**

| **Protein targeted** | **Host** | **Clone** | **Provider** | **Catalogue number** |
| --- | --- | --- | --- | --- |
| ADRP | Mouse | AP125 | Progen | 610102 |
| *Mitochondrial marker* | Mouse | MTC02 | Abcam | Ab3298 |
| Calreticulin | Mouse | FMC75 | Abcam | Ab22683 |
| Catalase | Rabbit | EPR1928Y | Abcam | Ab76110 |
| HCV core | Mouse | C7-50 | Axxora | ALX-804-277 |
| β-cytoplasmic actin | Mouse | C4 | Chemicon | MAB1501 R |

**(D) Secondary antibodies:**

| **Protein targeted** | **Host** | **Provider** | **Catalogue number** |
| --- | --- | --- | --- |
| HRP-conjugated anti-mouse | Goat | Biorad | 170-6516 |
| HRP-conjugated anti-rabbit | Goat | Biorad | 170-6515 |
| Alexa488-conjugated anti-mouse | Goat | Jackson |  |

**(E) Plasmids and HCV constructs :**

| **Plasmid insert/name** | **Backbone** | **Source** | **Reference** |
| --- | --- | --- | --- |
| HCV core | 2K7 | Invitrogen (Switzerland) | (Clement et al., 2010) |
| GFP | 2K7 | Invitrogen (Switzerland) | (Clement et al., 2010) |

**(F) Lipid standards :**

| **Name** | **Provider** | **Catalogue number** |
| --- | --- | --- |
| DLPC 12:0/12:0 | Avanti Polar Lipids Inc | 850335 |
| PE 17:0/14:1 | Avanti Polar Lipids Inc | PE31:1, LM-1104 |
| PI 17:0/14:1 | Avanti Polar Lipids Inc | PI31:1, LM-1504 |
| PS 17:0/14:1 | Avanti Polar Lipids Inc | PS31:1, LM-1304 |
| C14:0 Cardiolipin | Avanti Polar Lipids Inc | 710332P |
| C17:0 Ceramide | Avanti Polar Lipids Inc | 860517 |
| C12:0 SM | Avanti Polar Lipids Inc | 860583 |
| Glucosyl C8:0 Cer | Avanti Polar Lipids Inc | 860540 |
| Ergosterol | Fluka |  |

**(G) Other reagents :**

| **Name** | **Provider** | **Catalogue number** |
| --- | --- | --- |
| cOmplete *Protease Inhibitor* Cocktail | Roche | \| [04693116001](http://www.roche-applied-science.com/shop/en/global/products/complete-3271352-1) \|  \| \| --- \| --- \| |
| Miglustat | Tocris Bioscience | 3117 |
| Myriocin | Sigma | M1177 |
| TMP-153 | Santa Cruz | sc-200649 |
| Lipofectamin 2000 | Invitrogen | 11668-027 |
| ECL advance | Amersham | RPN2135 |
| Oil Red O (ORO) | Sigma | 75087 |
| Oleic acid | Sigma | O1008-5G |
| TG kit | Roche/Hitachi | 12016648-122 |
| Cholesterol/cholesteryl ester quantitation kit | Calbiochem | 428901-1 |
| MTBE | Fluka |  |
| Methylamine (33 % in absolute ethanol) | Sigma |  |
| HPLC grade chloroform and LC-MS grade ammonium acetate | Fluka |  |
| LC-MS grade water | Biosolve |  |

**References**

Clement, S., Pascarella, S., Conzelmann, S., Gonelle-Gispert, C., Guilloux, K. & Negro, F. (2010). The hepatitis C virus core protein indirectly induces alpha-smooth muscle actin expression in hepatic stellate cells via interleukin-8. *J Hepatol* **52**, 635-43.

Erez-Roman, R., Pienik, R. & Futerman, A. H. (2010). Increased ceramide synthase 2 and 6 mRNA levels in breast cancer tissues and correlation with sphingosine kinase expression. *Biochem Biophys Res Commun* **391**, 219-23.

Hoeferlin, L. A., Fekry, B., Ogretmen, B., Krupenko, S. A. & Krupenko, N. I. (2013). Folate stress induces apoptosis via p53-dependent de novo ceramide synthesis and up-regulation of ceramide synthase 6. *J Biol Chem* **288**, 12880-90.

Mullen, T. D., Spassieva, S., Jenkins, R. W., Kitatani, K., Bielawski, J., Hannun, Y. A. & Obeid, L. M. (2011). Selective knockdown of ceramide synthases reveals complex interregulation of sphingolipid metabolism. *J Lipid Res* **52**, 68-77.

Read, S. A., Tay, E., Shahidi, M., George, J. & Douglas, M. W. (2014). Hepatitis C virus infection mediates cholesteryl ester synthesis to facilitate infectious particle production. *J Gen Virol* **95**, 1900-10.

Sastry, L., Johnson, T., Hobson, M. J., Smucker, B. & Cornetta, K. (2002). Titering lentiviral vectors: comparison of DNA, RNA and marker expression methods. *Gene Ther* **9**, 1155-62.
